# Development of multi-drug resistance to anticancer drugs in HepG2 cells due to MRP2 upregulation on exposure to menthol

**DOI:** 10.1371/journal.pone.0291822

**Published:** 2023-09-21

**Authors:** Katsuhito Nagai, Mayuko Tamura, Ryuga Murayama, Shuhei Fukuno, Takuya Ito, Hiroki Konishi

**Affiliations:** 1 Laboratory of Clinical Pharmacy and Therapeutics, Faculty of Pharmacy, Osaka Ohtani University, Tondabayashi, Japan; 2 Laboratory of Natural Medicines, Faculty of Pharmacy, Osaka Ohtani University, Tondabayashi, Japan; PLOS ONE, UNITED KINGDOM

## Abstract

**Background:**

Menthol exerts relaxing, antibacterial, and anti-inflammatory activities, and is marketed as a functional food and therapeutic drug.

**Aim:**

In the present study, the effects of menthol on the expression of multidrug resistance associated protein 2 (MRP2) and its association with the cytotoxicity of epirubicin (EPI) and cisplatin (CIS) were examined using HepG2 cells.

**Methods:**

The expression levels of target genes were examined by real-time PCR. The intracellular concentration of incorporated EPI was measured by high-performance liquid chromatography. Cell viability was evaluated by MTT analysis.

**Results:**

The expression of MRP2 mRNA was increased by exposing HepG2 cells to menthol for 24 hr. Consistent with a previous report suggesting an inverse correlation between MRP2 and Akt behavior, increased expression of MRP2 was also observed on suppression of the Akt function. Intracellular accumulation of EPI was significantly decreased by exposure of HepG2 cells to menthol, and a significant decrease in the intracellular concentration of EPI remaining was observed in HepG2 cells exposed to menthol. The decreased intracellular accumulation of EPI was significantly suppressed by treatment with MK-571, but not verapamil. Both EPI and CIS exerted cytocidal effects on HepG2 cells, but the decrease in cell viability was significantly attenuated by 24-hr menthol pre-exposure.

**Conclusion:**

These results demonstrate that menthol causes hepatocellular carcinoma to acquire resistance to anticancer drugs such as EPI and CIS by MRP2 induction.

## Introduction

Hepatocellular carcinoma (HCC) is a serious malignancy worldwide, with increasing incidence and mortality over time [1]. Currently, approximately 80% of HCC patients are diagnosed at an advanced stage and are not amenable to surgical resection. Transarterial chemoembolization is currently believed to be the effective treatment for patients with advanced HCC, and epirubicin (EPI) and cisplatin (CIS) are widely utilized as anti-HCC agents [2, 3]. However, patients undergoing chemotherapy may increasingly develop multi-drug resistance (MDR) against anticancer agents, which is responsible for a major obstacle to HCC treatment. In addition, the intake of specific supplements can lead to MDR to anticancer agents [4]. There are a variety of mechanisms underlying MDR, one of which is the reduction in intracellular concentration associated with increased efflux of anticancer agents [5, 6]. ABC transporters such as the P-glycoprotein (P-gp) and multidrug resistance-associated protein (MRP) families play essential roles in this type of MDR [7, 8].

Menthol is a naturally occurring compound found in plants of the *Mentha genus*, commonly known as mint. Menthol is commercially available as a supplement and therapeutic drug because of several beneficial biological properties [9, 10]. Our previous study demonstrated that menthol causes hepatocellular carcinoma HepG2 cells to acquire resistance to doxorubicin (DOX) by increasing its extracellular efflux through the upregulation of P-gp [11]. However, whether the expression of other ABC transporters is affected by menthol exposure remains unclear. Recently, it was reported that suppression of the Akt signal following menthol exposure contributes to neuroprotection from inflammatory damage [12]. In light of experimental evidence that *Calculus Bovis*, a traditional Chinese medicine, could alleviate liver injury and up-regulate the expression of MRP2 in 17α-ethynylestradiol-induced cholestasis by a mechanism involving inhibition of the Akt signaling pathway [13], it is conceivable that MRP2 is induced following menthol exposure.

In the present study, using HepG2 cells, we examined the effects of menthol on the expression of MRP2 which markedly contributes to MDR of HCC [14]. Furthermore, the change in cytotoxicity of EPI and CIS was investigated following exposure to menthol.

## Materials and methods

### Chemicals

EPI hydrochloride and CIS were obtained from Sigma-Aldrich Co. (St. Louis, MO, USA). Menthol was purchased from Cosmo Bio Co., Ltd. (Tokyo, Japan). Dimethyl sulfoxide (DMSO) was from Fujifilm Wako Pure Chemical Co. (Osaka, Japan). HepG2 cells were obtained from the American Type Culture Collection (Manassas, VA, USA). All other reagents were of commercial or analytical grade and required no further purification.

### Cell culture

HepG2 cells were maintained in Dulbecco’s modified Eagle’s medium (Fujifilm Wako Pure Chemical Co.) containing 10% fetal bovine serum (Biowest USA, Riverside, MO, USA) at 37°C under a humidified atmosphere of 5% CO_2_ in air.

### Experimental design

For real-time PCR, HepG2 cells were exposed to either menthol or an Akt inhibitor for 24 hr. For the transport assay, HepG2 cells were exposed to menthol for 24 hr. The reagents used were dissolved in DMSO. Control cells were exposed to vehicle alone for the same time.

For the evaluation of cell viability, HepG2 cells were divided into four experimental groups, designated as: anticancer drug (EPI or CIS), menthol, anticancer drug plus menthol, and control groups. In the anticancer drug group, the cells were exposed to vehicle alone for 24 hr, followed by exposure to anticancer drugs for 24 hr. In the menthol group, the cells were consecutively incubated with menthol for 48 hr. In the anticancer drug plus menthol group, 24-hr simultaneous exposure to anticancer drugs and menthol was conducted 24 hr after addition of menthol. Control cells were exposed to vehicle alone for the same time.

### Real-time quantitative PCR

The total RNA fraction was extracted with ISOGEN (NIPPON GENE Co., LTD, Tokyo, Japan) and the GenElute Mammalian Total RNA Miniprep kit (Sigma-Aldrich Co.). Real-time quantitative PCR was performed using SYBR green as the fluorescent dye (Toyobo Co., Ltd., Osaka, Japan), following reverse transcription. The conditions for denaturation, annealing, and extension reactions in the PCR process were 95°C for 10 sec, 57°C for 10 sec, and 72°C for 30 sec, respectively. 18S-ribosome was used as an internal control. The base sequences of the MRP2 primer were 5’-CCTAGACAACGGGAAGATT-3’ (5’ primer) and 5’-CCATAAAGTAAAAGGGTCCAG-3’ (3’ primer). The base sequences of the 18S-ribosome primer were 5’-CGTCTGCCCTATCAACTTTCG-3’ (5’ primer) and 5’-CGTTTCTCAGGCTCCCTCT-3’ (3’ primer). The mRNA levels were quantified based on standard curves, and the results are expressed relative to control values, which were given an arbitrary value of 1.

### Transport assay

After pre-incubation of HepG2 cells with Hanks’ balanced salt solution (HBSS) for 10 min in the presence or absence of 100 μM MK-571 or verapamil, the cells were exposed to EPI at a final concentration of 5 μM for certain amounts of times. The transport reaction was stopped by the addition of ice-cooled excess phosphate-buffered saline (PBS). The cells were lysated using ultrapure water. In the efflux experiment, HepG2 cells had been loaded with EPI in HBSS for 30 min, and then were incubated in drug-free HBSS for 3 min following washing twice with ice-cold PBS. The procedure after the reaction stop was performed in the same manner as described above.

### Assay procedure

After the addition of 50 μL of 2 μg/mL daunorubicin (Sigma-Aldrich Co.), an internal standard (IS), 75 μL of 4% zinc sulfate, and 75 μL of methanol to a 100-μL lysis sample, the mixture was vortexed and centrifuged at 5,000 g for 10 min. One hundred microliters of supernatant were injected into the high-performance liquid chromatography (HPLC) system. The analytes were separated on an RP-18 GP II column (150 × 4.6 mm; Kanto Chemical Co., Inc., Tokyo, Japan) using a mixture of 15 mM acetate buffer (pH 2.5) and acetonitrile (v/v, 2:3) as the mobile phase. The mobile phase was delivered at 0.7 mL/min, and the column effluent was monitored with an excitation wavelength of 470 nm and an emission wavelength of 550 nm. The concentration of EPI was quantified using the peak area ratio compared with the IS. The protein concentration was measured based on the Bradford method and applied to the estimation of the intracellular amount of EPI.

### Cell viability

On the 3rd day, cell viability was evaluated by measuring mitochondrial enzyme activity for reducing MTT to corresponding formazan dye according to our previous report [11]. Cell viability is expressed relative to control values, given an arbitrary value of 100.

### Statistical analysis

Data are expressed as means ± SD. Differences between the means of two groups were evaluated using Student’s unpaired *t*-test. The significance of differences between control and test values was determined using Dunnett’s test or the two-tailed multiple *t*-test with Bonferroni correction following one-way analysis of variance. Differences with a *p*-value of 0.05 or less were considered significant.

## Results

### Effect of menthol and Akt inhibitor on the expression of mRNA for MRP2

**Fig 1:**
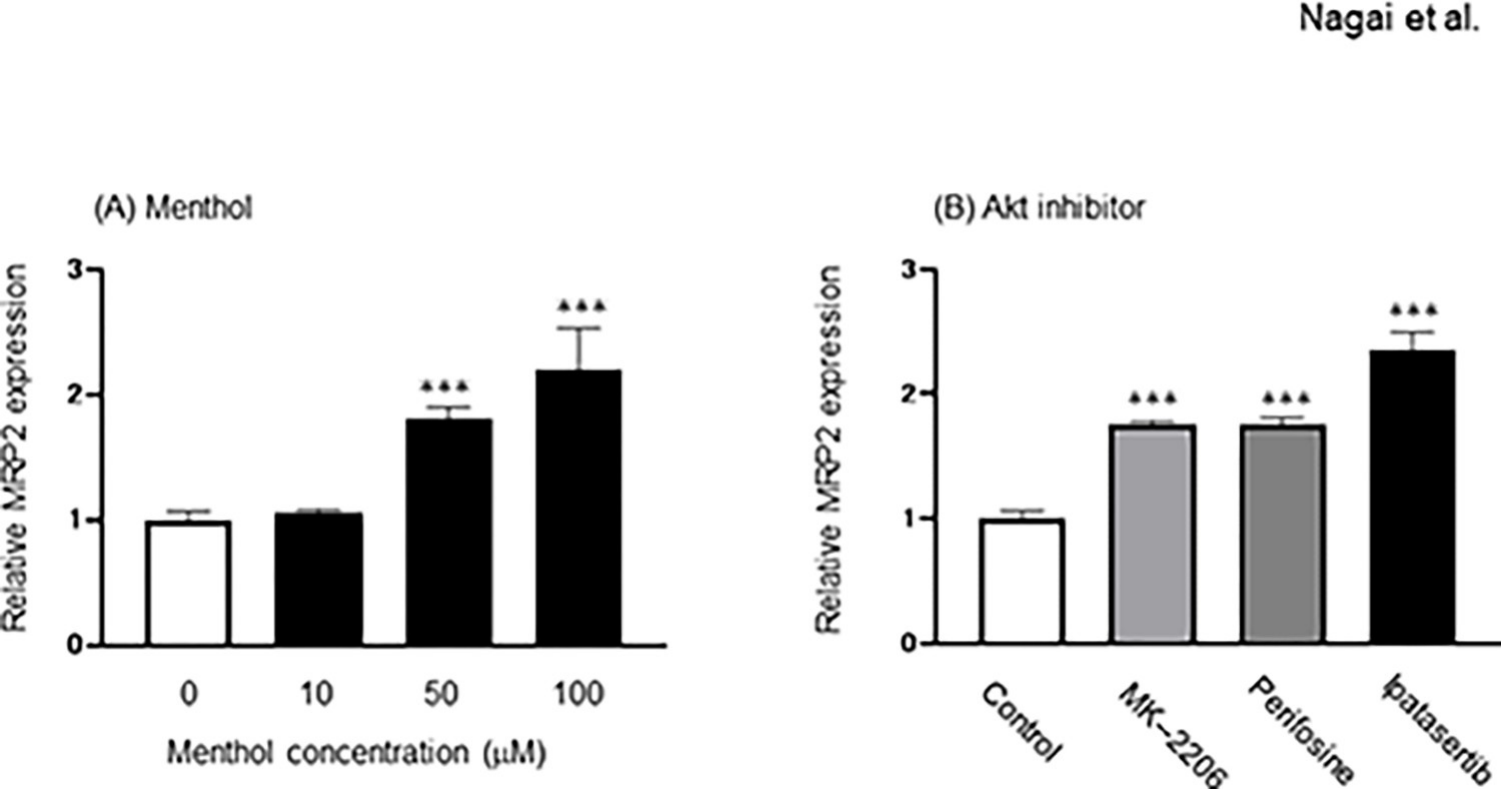
Effects of menthol and Akt inhibitors on the gene expression of MRP2. HepG2 cells were exposed to 10–100 μM menthol, 5 μM MK-2206, 40 μM perifosine, or 40 μM ipatasertib for 24 hr. Total RNA was extracted and purified from HepG2 cells, and the expression level of MRP2 was measured by real-time PCR. The results represent the mean ± SD of three independent experiments. ***: *p*<0.001 (*ver*. Control group).

The effect of menthol and Akt inhibitors on gene expression of MRP2 was examined (Fig 1). While 10 μM menthol had no significant effect on expression of MRP2, significantly higher expression of MRP2 was observed in HepG2 cells exposed to menthol at concentrations of 50 and 100 μM (Fig 1A). The expression of MRP2 mRNA was significantly increased by exposure of HepG2 cells to 5 μM MK-2206, 40 μM perifosine, or 40 μM ipatasertib, which are Akt inhibitors (Fig 1B).

### Effects of menthol on the activity of EPI transport

**Fig 2:**
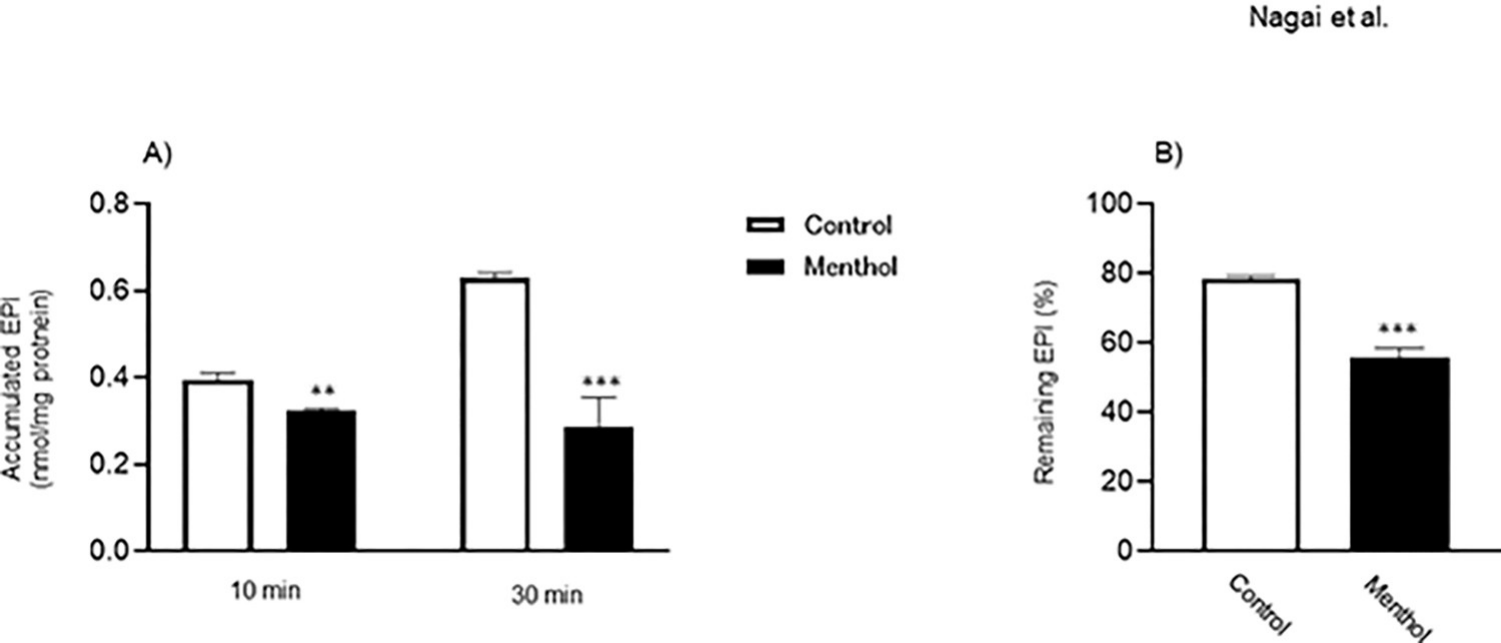
Effect of menthol on intracellular accumulation and efflux of EPI. HepG2 cells were exposed to 100 μM menthol for 24 hr. (A) Cells were preincubated for 10 min at 37°C and then incubated with 5 μM EPI for 10 or 30 min. (B) After the cells had been loaded with 5 μM EPI for 30 min at 37°C, they were incubated in drug-free HBSS for 3 min at 37°C. The results represent the mean ± SD of three independent experiments. **: *p*<0.01 (*ver*. Control group), ***: *p*<0.001 (*ver*. Control group).

**Fig 3:**
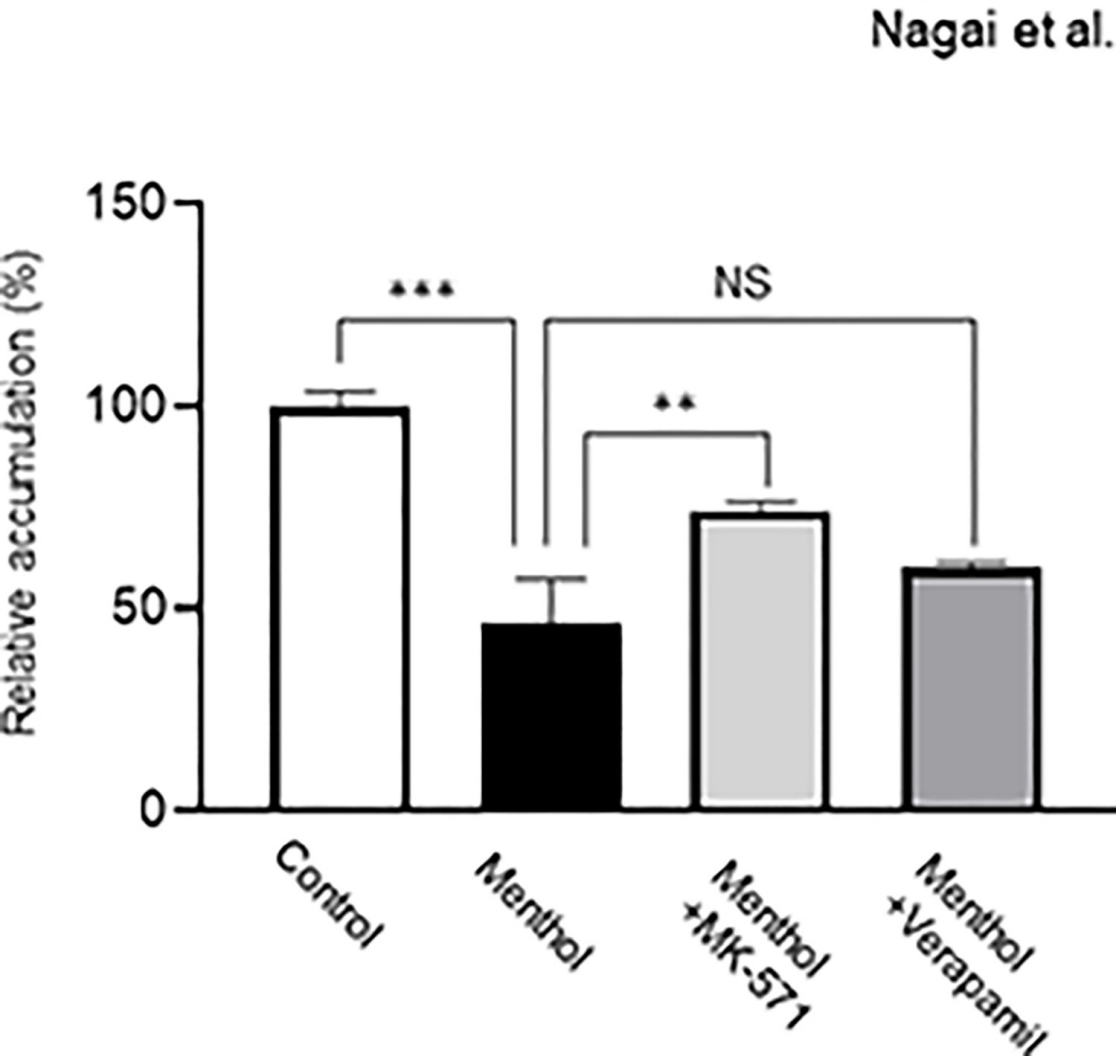
Effect of MK-571 and verapamil on decreased intracellular accumulation of EPI after menthol exposure. HepG2 cells were exposed to 100 μM menthol for 24 hr. Cells were preincubated for 10 min at 37°C with or without 100 μM MK-571, and 100 μM verapamil, and then incubated with 5 μM EPI for 30 min under the same conditions as for preincubation. The results represent the mean ± SD of three independent experiments. **: *p*<0.01, ***: *p*<0.001.

In the examination of EPI transport, its intracellular amount transported into HepG2 cells in the menthol-exposed group was significantly lower than that in the control group (Fig 2A). In the efflux experiment, the intracellular level of EPI remaining in HepG2 cells was significantly decreased by menthol exposure (Fig 2B). As shown in Fig 3, the decreased intracellular EPI accumulation of EPI in HepG2 cells exposed to menthol was almost cancelled by treatment with MK-571, but not verapamil.

### Effects of menthol on the cytotoxicity of EPI and CIS

**Fig 4:**
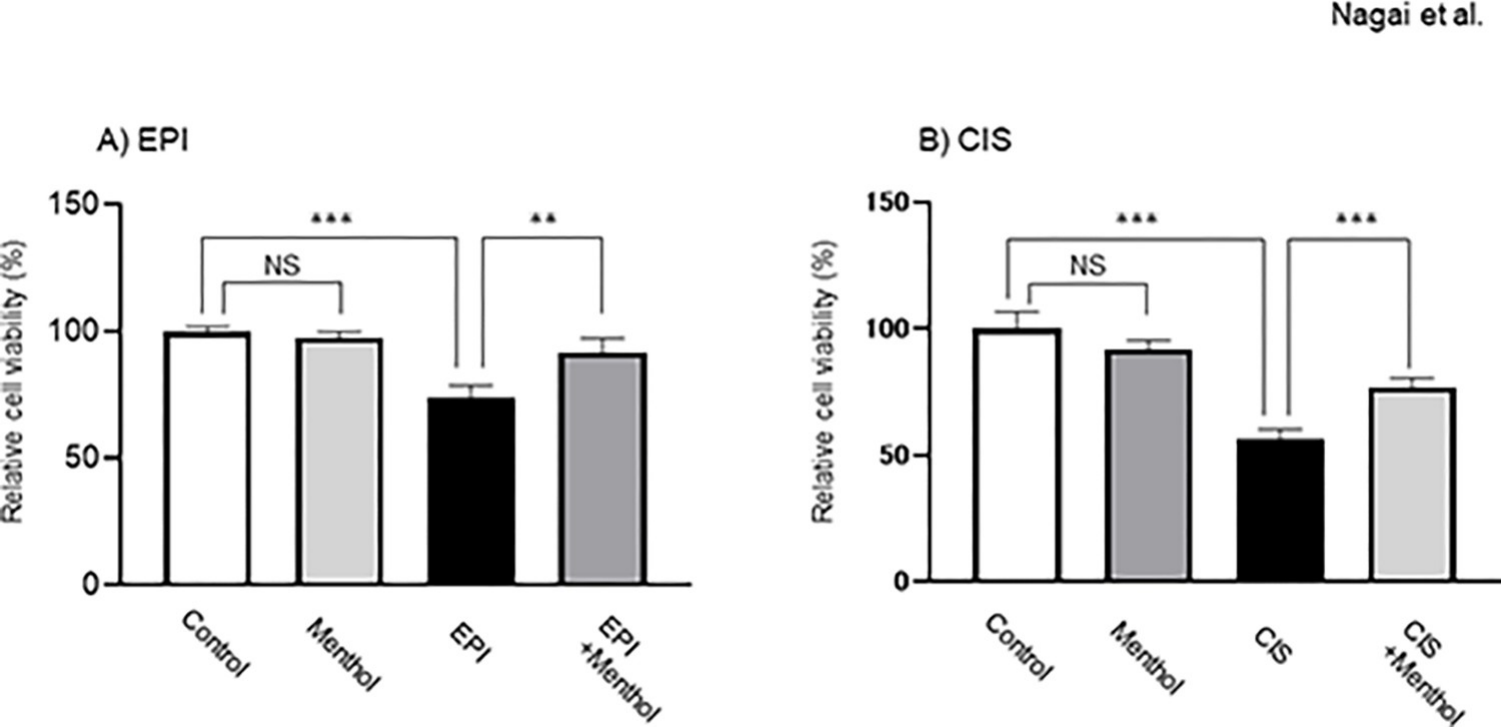
Effects of menthol on cytotoxicity of EPI and CIS. The cells were exposed to 5 μM EPI or 80 μM CIS 24 hr after treatment with 100 μM menthol, and cell viability was then evaluated by the MTT method 24 hr later. The results are shown as means ± SD of three independent experiments. **: *p*<0.01, ***: *p*<0.001, NS: not significant.

The viability of HepG2 cells was decreased in the presence of EPI or CIS, but the decreases were significantly attenuated by exposure to menthol in advance (Fig 4). Menthol alone had no effect on viability of HepG2 cells (Fig 4).

## Discussion

Induction of some MRP families is responsible for MDR to anticancer drugs and is a major obstacle to the conduction of cancer chemotherapy [15, 16]. MRP1 (ABCC1), the first cloned member of this family, confers resistance to a variety of drugs such as anthracyclines and vinca alkaloids [17]. However, no significant expression of MRP1 mRNA was detected in human liver [18]. Resistance to CIS and anthracyclines was shown in MRP2-overexpressing cells constructed by molecular techniques [19]. MRP2 was reported to be expressed on the plasma membrane of all HCCs used in this study, while MRP1 was expressed only in some HCCs and localized to the intracellular membrane [14]. Therefore, it is likely that MRP2 rather than MRP1 is much deeply implicated in HCC resistance. In the present study, we investigated using HepG2 cells the effects of menthol on MRP2 expression and the cytotoxicity of anticancer drugs serving as substrates of MRP2.

It was confirmed that gene expression of MRP2 in HepG2 cells was increased by exposure to menthol as was when the Akt function was suppressed in the presence of its inhibitor. These results support experimental observation in studies using animals that neuronal Akt pathway was suppressed after menthol administration and whereby the expression of MRP2 was inversely related to the signal response to the Akt pathway [12, 13]. In HepG2 cells exposed to menthol, the intracellular accumulation of EPI was significantly reduced, indicating the marked contribution of menthol to enhanced efflux of the drug. The previous study demonstrated that higher expression of P-gp, MRP1, and MRP2 was observed in Hela cells exposed to EPI [20]. The gene expression of these transporters in cancer cells can be decreased by various molecular techniques, which causes an increase in intracellular EPI accumulation [21–25]. Furthermore, the cytotoxic effect of EPI on diffuse large B-cell lymphoma cells was enhanced by NF-κB pathway-mediated control of P-gp expression [26]. These findings suggested the possible involvement of P-gp, MRP1, and MRP2 in efflux of EPI. However, our recent study demonstrated that MRP families were markedly involved in EPI efflux rather than P-gp [27]. Furthermore, in the present study, the enhanced function of MRP2 by menthol exposure can be explained by the increased intracellular EPI level in the presence of MK-571 (MRP2 inhibitor), but not verapamil (P-gp inhibitor), in menthol-exposed HepG2 cells. Based on these findings, we strongly consider that MRP2 plays a central role as a determinant of the intracellular EPI concentration by acting as an efflux pump, although it is susceptible to induction by menthol.

HepG2 cells were shown to be sensitive to EPI, but decreased viability was significantly attenuated by pre-exposure to menthol. As the cytocidal efficacy of anthracyclines was likely to be correlated with the intracellular accumulation of the drugs [27], it is strongly suggested that induction of MRP2 following menthol exposure is a cause of the reduced cytotoxicity of EPI. To confirm that menthol is generally capable of producing MRP2-mediated MDR, it was necessary to evaluate whether the cytotoxic effect of other anticancer drugs would also be attenuated by menthol. In previous studies, the observed resistance to CIS could not be explained by induction of P-gp expression [28]. Active efflux of glutathione-conjugated CIS was reported to be mediated by MRP2 [29], which was supported by evidence that intracellular glutathione levels were related to CIS cytotoxicity [30]. The observation is suggestive of the possible involvement of efflux mediated by MRP2 in a CIS resistance. Furthermore, the previous studies have clarified that reduction in intracellular accumulation of CIS is associated with increased levels of MRP2 mRNA, which resulted in resistance to CIS [31]. In the present study, cytocidal potency of CIS was also attenuated by pre-exposing HepG2 cells to menthol, suggesting that the development of MDR attributed to menthol exposure is common across anticancer drugs serving as substrates of MRP2, leading to serious issues associated with therapeutic failure.

Peppermint oil, which contains menthol as principal ingredient, has been shown to be useful in treating gastroesophageal reflux and irritable bowel syndrome (IBS) [10, 32, 33]. The usual daily dose of peppermint oil for the treatment of IBS is about 1.1 g. The serum concentrations of menthol have been determined to reach 10 μM when 36 mg peppermint oil capsules were orally administered to healthy male volunteers [34]. In this regard, when peppermint oil capsules are used for the treatment of IBS, serum levels of menthol can reach 100 μM due to efficient intestinal absorption with over 70% bioavailability [35], which can lead to the failure of HCC treatment through upregulation of MRP2 in clinical settings. In addition to EPI and CIS, MRP2 contributes to the efflux of many structurally unrelated chemotherapeutic drugs such as vincristine [7]. Therefore, medical staff should advise HCC patients receiving anticancer drugs not to take menthol-rich supplements to reduce the risk of MDR.

## Conclusion

The present study demonstrated that menthol exposure carries a risk of developing MDR in HepG2 cells due to the upregulation of MRP2. Our study will help to promote the successful treatment of HCC using anticancer drugs.
